# A perspective on magnetic ionic liquids as multifunctional platforms for drug delivery and biomedical applications

**DOI:** 10.1186/s11671-026-04546-1

**Published:** 2026-04-05

**Authors:** Ashwini Kumar Mishra, Sweta Acharya, Ankit Jain

**Affiliations:** 1 Department of Pharmaceutics, School of Pharmacy and Technology Management, SVKM’s NMIMS Deemed-to-be University, Shirpur, Maharashtra 425405 India; 2 Industrial Research Laboratories, Department of Pharmacy, Birla Institute of Technology and Science, Pilani Campus, Pilani, Rajasthan, 333031 India

**Keywords:** Magnetic ionic liquids, Drug delivery, Biomedical applications, Paramagnetic properties, Computational methods

## Abstract

The demand for advanced field-responsive materials has positioned magnetic ionic liquids (MILs) as a transformative class of tunable fluids that bridge materials science and biomedicine. Initially valued for magnetically induced behaviors, MILs excel in surface activity, solubility enhancement (up to 39,000-fold for poorly soluble drugs), colloidal stabilization, and polymer-surfactant interactions at interfaces. Recent computational advances, Density Functional Theory (DFT) for electronic structures and ion reactivity, alongside Molecular Dynamics (MD) simulations, predict properties across timescales, enabling design-driven synthesis of low-viscosity, high-magnetic-moment formulations with thermal stability to 345 °C. In biomedicine, MILs promise targeted drug delivery, NIR-fluorescent theranostics, and dual-mode MRI contrast via paramagnetic chelates, with 2025 breakthroughs in magnet-guided tumor nanocomplexes outperforming traditional nanoparticles. Their significance lies in their use as stimuli-responsive platforms for oncology, neurology, and antimicrobial therapies, thereby enhancing bioavailability and enabling green, recyclable pharmaceutical processes. Yet, critical gaps persist. Fe-based MILs suffer from hydrolysis and reproducibility issues, while Co/Mn variants raise toxicity concerns, limiting biocompatibility, in vivo biodistribution studies, and pharmacokinetics data. Scalability hurdles, such as high costs, non-standardized characterization, and inadequate models for field effects on transport, hinder clinical translation. This perspective critically reflects on the evolution of MIL research, integrating experimental and computational advances, and underscores the need for design-driven synthesis, standardised characterisation, and application-oriented strategies. By highlighting achievements, addressing key challenges, and mapping future directions, we aim to stimulate cross-disciplinary dialogue and accelerate the translation of MILs into next-generation technological and biomedical platforms.

## Introduction

Drug delivery encompasses diverse processes, including formulation, production, storage, and the development of advanced technologies to precisely transport pharmaceutical compounds to their intended site of action. Achieving optimal pharmacological effects requires careful consideration of production methods, routes of administration, target-site orientation, and metabolism to improve efficacy, safety, compliance, and therapeutic outcomes. Conventional strategies rely on modifying drug pharmacokinetics through carriers, fillers, and devices, yet challenges such as poor bioavailability and the need for controlled, site-specific release remain unresolved, especially for hydrophobic drugs [1]. Nanocarrier-based systems, including lipid, polymeric, metallic, graphene, and nanocrystal formulations, have attempted to address these limitations [2–4]. Among them, metal-based nanocarriers have shown promising effects in various applications, including colloidal particles, nanomicelles, nanoemulsions, nanocapsules, and nanogels. Their unique nanoscale properties enhance the solubility and therapeutic efficacy of small-molecule drugs, as well as genetic materials and macromolecular polypeptides. This is particularly beneficial in treating cancer, diabetes, and cardiovascular diseases. However, their use is limited by the toxicity of certain organic solvents, such as dichloromethane (DCM), dimethyl sulfoxide (DMSO), acetonitrile (ACN), acetone, ethanol, and hexane [5–9].

In this Perspective, we argue that excipient-free delivery approaches using ionic liquids (ILs) may overcome these limitations. ILs are molten salts with unique physicochemical features, including low vapour pressure, chemical stability, and non-combustibility. These features have made them attractive across disciplines, from electrochemistry and catalysis to biomedicine. They consist of bulky cations, such as imidazolium, pyrrolidinium, or phosphonium, and various small organic or inorganic anions, including tetrafluoroborate (BF_4_^−^), bis(trifluoromethylsulfonyl)imide [(CF_3_SO_2_)_2_N]^−^, acetate (CH_3_COO^−^), and dicyanamide [N(CN)₂]⁻ or even halides [10–12]. Within this class, Magnetic Ionic Liquids (MILs) represent a specialised subcategory incorporating paramagnetic ions from transition metals or lanthanides, imparting responsiveness to external magnetic fields [13]. Beyond their fundamental magneto-responsive properties, MILs have demonstrated biomedical potential in anticancer therapy, tissue engineering, biomaterial synthesis, and drug delivery, where they improve drug bioavailability and enable target-specific release [14, 15]. Their applications extend to DNA extraction, protein and gene delivery, gas capture, and sensing technologies, with implications for cancer diagnosis and therapy [16]. Conjugating MILs with photodynamic dyes further opens new avenues for theragnostic, combining imaging and treatment within a single platform [17]. Critically, MILs offer unique opportunities beyond conventional nanocarriers; they can act simultaneously as drug carriers and nanoprobes, enable magnetically guided or light/heat-triggered release, and be functionalized with ligands or recognition molecules for active targeting [18–20]. The interest in ILs in this studystems from MILs’ capacity to infiltrate tissues via blood vessels and selectively target specific cells via recognition molecules, enabling active targeting. Examples include imidazolium-based MILs delivering genetic materials in chemotherapy [21, 22], di-cationic MILs tuned with hetero-anionic combinations [23], and lanthanide complexes demonstrating both magnetic and luminescent activity [24, 25]. In 2009, Li et al. reported the synthesis of chiral MILs from amino acids that combine stereochemical and magnetic properties, highlighting opportunities for the design of multifunctional therapeutic platforms [26].

MIL research must progress beyond proof-of-concept demonstrations toward biocompatible, tunable, and application-oriented systems. Their dual roles as responsive drug carriers and functional nanomaterials position them at the intersection of the pharmaceutical sciences, materials engineering, and biomedicine. Future research should prioritize: (i) Design-driven synthesis informed by computational modelling; (ii) Systematic evaluation of toxicity and stability; (iii) Expanding biomedical applications to include protein stabilization, macromolecule drug delivery, biosensing, and antibacterial functions; and (iv) Integrating MILs with nanocarrier functionalization strategies for enhanced precision. Computational approaches, especially DFT and MD simulations, will be essential in elucidating complex ion interactions, predicting stability, and guiding rational design.

Many reviews address ILs and MILs, covering physicochemical properties, synthesis and characterization, MILs as solubilizers for poorly soluble medicinal agents, antimicrobial effects, and their use in separation and environmental chemistry. Although these published articles provide a descriptive overview of MILs across various classes, they have limited impact on translational findings due to design constraints, poor performance metrics, and inconclusive verdicts regarding biomedical use. Compared with prior studies, the current manuscript is not limited to a compilation of prior studies and provides a concise analysis of MILs, focusing on their design-oriented and biomedical applicability. This review presents key considerations for scalability and clinical translation by addressing challenges such as ion selection, toxic effects, and the in vivo/in vitro stability of MILs. Through computational modelling, we proposed a future perspective: MILs are not only limited to substituting for nanomaterials; by understanding structure-function relationships, the chemistry of cation-anion interactions, and magnetic properties, they can be utilized for next-generation drug delivery and diagnostic applications. This Perspective does not merely review current findings; instead, it emphasizes the unique ability of MILs to address persistent challenges in drug delivery while opening new avenues in theranostics, biosensing, and materials science. By fostering interdisciplinary collaborations, MILs could transform from emerging laboratory curiosities into robust, next-generation platforms for pharmaceutical and biomedical innovation.

## Synthesis challenges in designing magnetic ionic liquids

A thorough understanding of strategies for synthesising MILs is crucial for recognising their potential and limitations. Unlike conventional ionic liquids, there is no universal method for producing MILs, and their physicochemical and magnetic properties must often be tailored by careful modification of cationic and anionic components. This complexity introduces several persistent challenges, including securing the requisite ferromagnetic properties, achieving kinetic and thermodynamic stability, and ensuring process sustainability. For instance, although the paramagnetic behaviour of iron-based MILs has been widely studied, their instability in aqueous solutions due to hydrolysis remains a significant drawback. To mitigate this, metals such as Au, Pd, Pt, Dy, Mn, Ni, Gd, and Ag have been incorporated to improve hydrolytic stability and introduce diverse paramagnetic behaviours through varied interactions such as hydrogen bonding, π–π stacking, and dispersive charge interactions [27–29].

In this context, design considerations are shifting toward metals with favourable electronic configurations and synthetic strategies that balance stability with functionality. For example, Mn-based systems (3d⁵) can form octahedral magnetic structures with carboxylates and N-donor macrocycle ligands, which are relatively straightforward to synthesise, less toxic, and economically favourable [30]. Similarly, early studies demonstrated the potential of 1-butyl-3-methylimidazolium tetrachloroferrate ([Bmim^+^][FeCl₄^–^]), synthesised via simple salt metathesis, to exhibit solubility in aqueous systems while maintaining paramagnetic properties [31, 32]. More recently, MILs have been explored as solvents for the extraction, separation, and purification of natural products, representing an effort to replace hazardous, low-yielding industrial methods with greener alternatives [33, 34]. While time-consuming processes and reliance on volatile organic solvents remain limitations, magnet-assisted phase separation, optimised pH/temperature control, and polymer-assisted aqueous systems are emerging as strategies for scalable and sustainable synthesis [35–38].

**Fig. 1:**
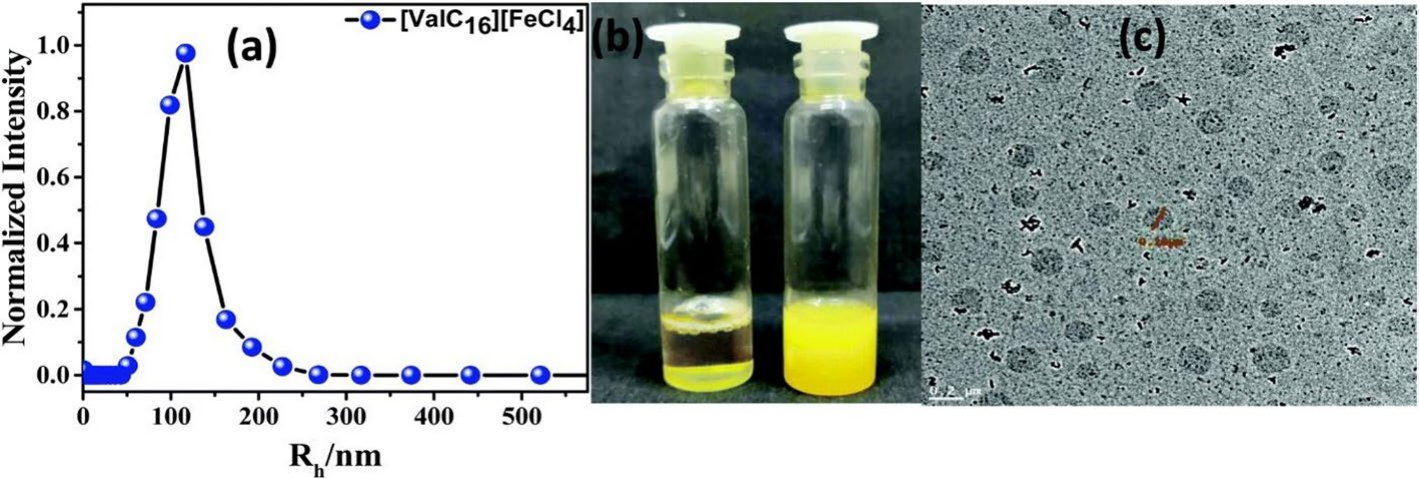
The (**a**) Dynamic light scattering graph of MIL rod-like micelles of 8 nm, which changes to vesicles of 95 nm, turning the solution turbid on the increase in size, is confirmed in (**c**) HR-TEM images. Adapted with permission from [39] under the Creative Commons Attribution 3.0 Unported Licence (https://creativecommons.org/licenses/by/3.0/)

New synthetic innovations highlight the versatility of MILs. For instance, magneto-responsive biomaterial hydrogels prepared from amino acid-based ionic liquids (Fig. 1(a)) demonstrate how MILs can be integrated into biocompatible gels with drug-delivery potential (see Fig. 1(b & c)) [39]. Oliveira et al. synthesised magnetic multifunctional spinel ferrite nanoparticles (MFe₂O₄) nanoparticles (M = Co, Fe, Mn, Ni) in ionic liquids, demonstrating the feasibility of producing size-controlled, superparamagnetic nanomaterials for biomedical and energy applications, with surface modifications that enable dispersibility in both polar and non-polar solvents [40]. Such approaches underscore the adaptability of MILs, but also point to the need for systematic frameworks to guide reproducibility and standardisation [41].

**Fig. 2:**
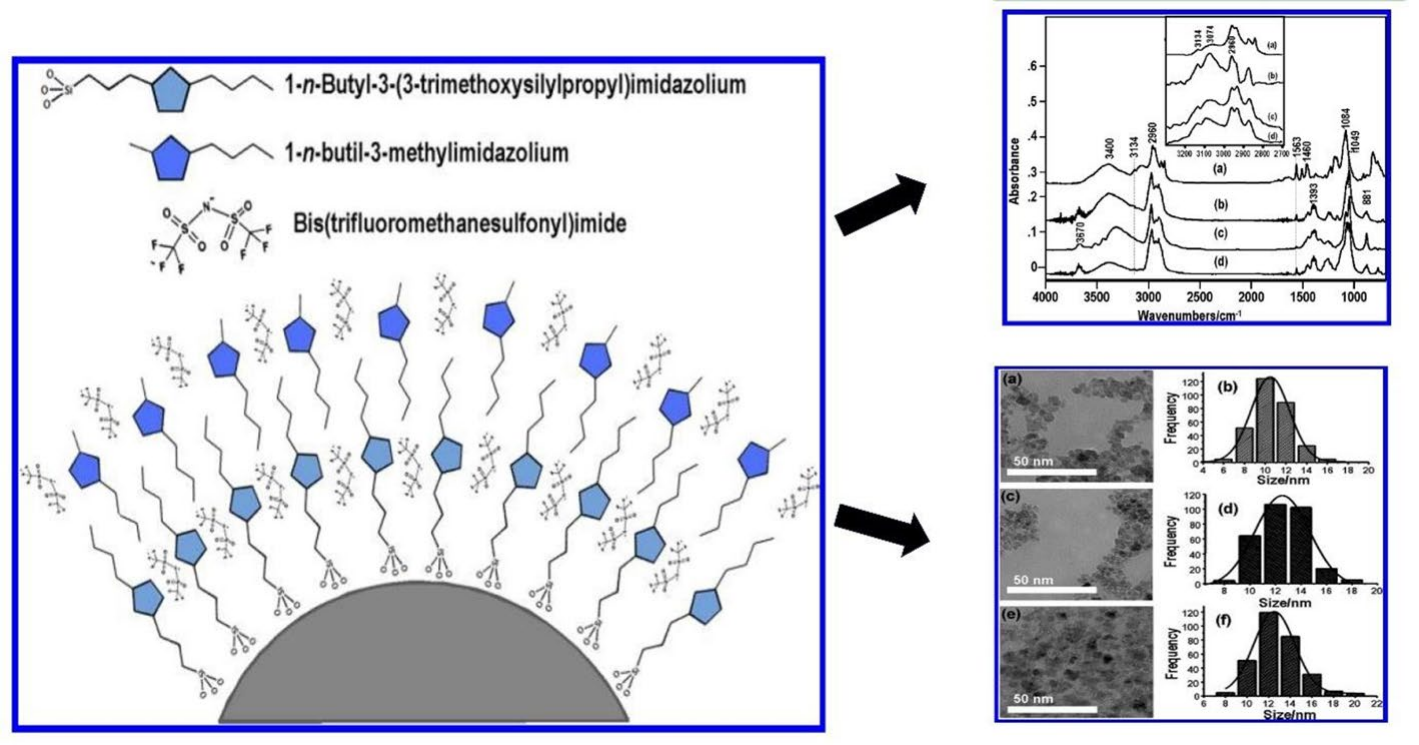
The protective layer formed by 1-n-butyl-3-methylimidazolium bis(trifluoromethanesulfonyl)imide on surface-modified magnetic nanoparticles via silanization, confirmed by FTIR-ATR, is spherical, with a mean diameter of 10–12 nm, as shown in TEM images and corresponding histograms. Adapted with permission from [50], Copyright © 2012, American Chemical Society

Recent advances in tailoring cationic and anionic components further highlight this complexity. Nacham et al.‘s work demonstrated that mono-, di-, and tricationic MILs can be generated from a tetrahalometallate inorganic anion such as [FeCl₃Br–] and related anions, producing systems with tuneable hydrophobicity and magnetic responsiveness. While such strategies improve yields and allow control of physicochemical properties, they often involve multi-step, labour-intensive procedures [42]. Similarly, Medeiros, Anderson, et al. developed the surface-modified nanoparticles using imidazolium-based ionic liquids (e.g., BMSPI.Cl), which have shown long-term stability due to supramolecular frameworks that mimic ionic liquid structures and minimise particle coagulation (see Fig. 2) [43].

A notable advance is the incorporation of paramagnetic components directly into cations (e.g., Fe(III) carboxylate complexes), providing flexibility to attach functional anions while preserving paramagnetism [31, 44–46]. Hydrophobic MILs produced via in situ metathesis further expand their potential applications by generating finely dispersed droplets with a large surface area, which are suitable for drug delivery systems [47, 48]. Finally, surface-active MILs based on environmentally friendly cations (e.g., tetramethylguanidinium, chiral amino acids, cholinium) combined with paramagnetic anions (e.g., [CoCl₄]²–, [CuCl₄]²–, [FeCl₄]–, [GdCl₆]³–) provide an avenue for designing systems that balance functionality with sustainability [49].

From a Perspective standpoint, while significant strides have been made in MIL synthesis, the field continues to face challenges in scalability, reproducibility, environmental impact, and control of physicochemical behavior. We believe the future of MIL design lies in: (i) Expanding beyond halide-based systems to sustainable, bio-inspired chemistries; (ii) Integrating computational modelling to predict stability and optimize component selection; (iii) Developing green, solvent-free, or magnet-assisted processes for large-scale synthesis; and (iv) Creating modular design principles for application-specific MILs. By addressing these directions, the synthesis of MILs can transition from a collection of experimental approaches to a rational, design-driven discipline, enabling their broader use in drug delivery, biomedicine, catalysis, and innovative material applications.

## Understanding MILs through new generation computational modelling

**Fig. 3:**
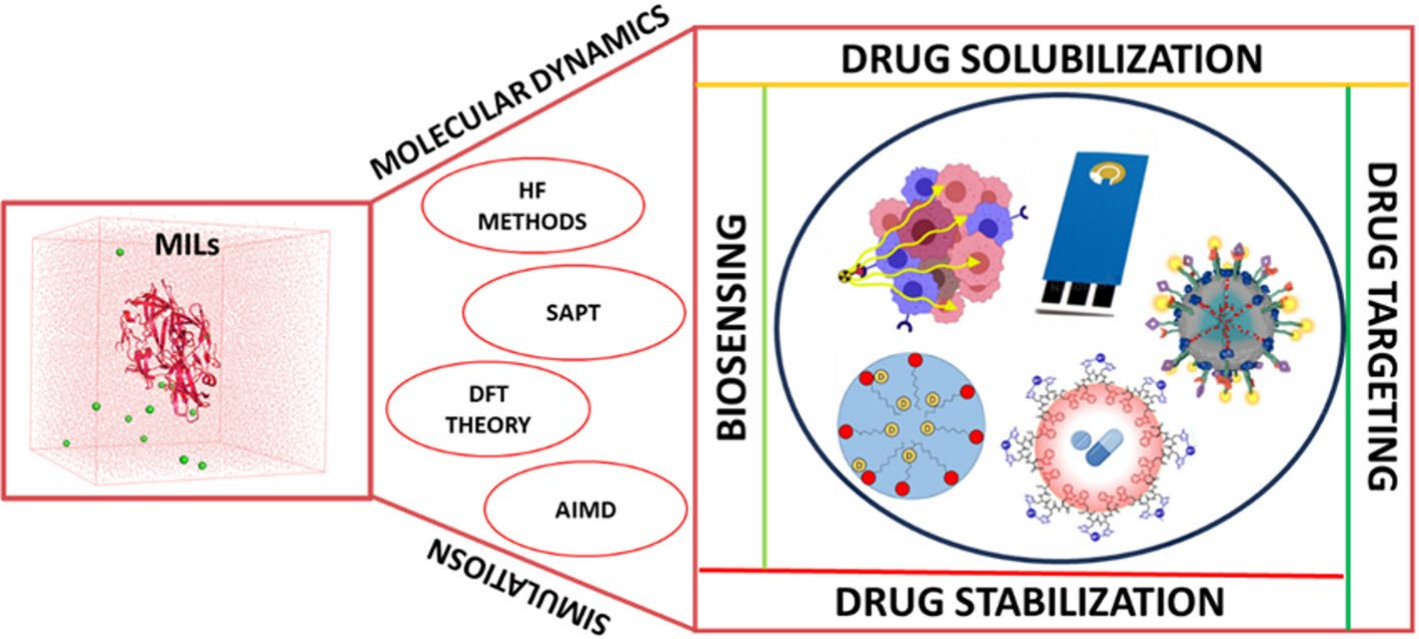
Use of new-generation computational modelling for designing MILs with desirable properties

The design and development of ionic liquids, particularly MILs, pose unique challenges due to the vast combinatorial space of cation-anion pairings and the inclusion of paramagnetic metal centres. Computational modelling has become indispensable, offering predictive insights that guide experimental design, minimise costly trial-and-error approaches, and accelerate translation toward biomedical applications. By enabling molecular-level interrogation of structure–property relationships, simulations provide a rational framework for identifying promising MIL candidates [51, 52]. Molecular dynamics (MD) simulations, ab initio quantum mechanical approaches, and density functional theory (DFT) have been applied to elucidate the electronic structures, non-covalent interactions, and physicochemical behaviour of MILs [51, 53, 54]. Energy decomposition analysis, such as symmetry-adapted perturbation theory (SAPT), has proven valuable for deconvoluting the contributions of hydrophobic, electrostatic, and London dispersion forces that govern ion-pair assembly and ultimately determine conductivity, viscosity, and solubility [55–58]. Recent computational research has focused on electronic structure calculations to investigate the properties of MILs (see Fig. 3). However, their open-shell nature poses additional hurdles to the existence of paramagnetic atoms. Modelling the electrical structure and magnetic properties of metal-organic frameworks, especially those containing many electrons, such as rare-earth metals, poses considerable challenges. The main goal in examining open-shell systems is to utilise a basis set that can precisely represent their charge density. Table 1 gives an overview of continuum-level materials simulations that help to study the behaviour of MILs. A diverse array of pyrrolidinium- and imidazolium-based ILs featuring alkyl chains was examined in conjunction with eight frequently utilised anions (BF_4_^−^, PF_6_^−^, mesylate, Cl^−^, Br^−^, tosylate, dicyanamide, and bis (trifluoromethane sulfonyl) imide) by the application of SAPT. As indicated in the cited studies, the interactions of steric repulsion, induction, electrostatic, and London forces were found to regulate the interionic arrangement in the examined ion pairs. Furthermore, SAPT calculations are widely used to develop force fields (FFs) with induced dipoles for computational models of MILs [56, 59]. Yet the open-shell electronic configuration of MILs remains a fundamental barrier. Paramagnetic ions introduce multiple electronic states, complicating wave function representation and challenging conventional closed-shell computational frameworks [60]. While quantum mechanical computations provide valuable static snapshots, they remain limited to small gas-phase systems. To address these limitations, hybrid approaches such as Car-Parrinello molecular dynamics (CPMD) and atomistic MD simulations are increasingly employed to capture dynamic processes [61, 62]. Car-Parrinello molecular dynamics (CPMD) integrates DFT with MD by treating electronic orbitals as dynamic variables alongside nuclear positions. In conventional Born-Oppenheimer DFT-MD (BO-MD), electronic minimization is performed at every stage. In contrast, CPMD overcomes this challenge, enabling faster simulations of dynamic phenomena in complex liquids such as ILs, including prospective MIL expansions. In a study by Bühl et al., IL [dmim]Cl was analysed using density-functional-based CPMD simulations at 438 K. A large amount of hydrogen bonding is evident in the liquid’s structure, as indicated by anisotropic spatial distributions and distinct partial radial distribution functions. While the theoretical cation−cation distribution shows poorer agreement, the cation−anion distribution simulated with the BP86 functional is qualitatively consistent with the structural model derived from neutron diffraction data for the liquid. It is clear from population analysis that anions transfer charge to cations, and that there are specific CH··· interactions. This bridges the static gaps of DFT by measuring interactions between lone pairs on the Cl atom and antibonding σ*CH orbitals, which describe Cl hydrogen bonds, substantially showing that CPMD can be a useful tool for studying MILs behaviour via transferable force fields [63].

Despite these advances, computational research on MILs remains sparse, largely due to inadequate, non-transferable force fields for diverse cation–anion families. Future directions must therefore focus on systematically developing validated FFs for broader structural classes, particularly paramagnetic anions, to enable predictive modelling of MILs in biologically relevant environments. Such progress will be pivotal in advancing MILs from conceptual materials to functional platforms for drug delivery, biosensing, and other innovative applications.

**Table 1:** An overview of Continuum-level materials simulations used to study the behaviour of MILs

MILs with their computational simulations	Types of interactions studied	Properties predicted	Ref.
DFT calculations in 1,3-dimethylimidazolium tetrabromoferrate ([Dimim] [FeBr_4_])	Anion-π interactions	Magnetic susceptibility, chemical reactivity, and electrical conductivity	[64]
DFT calculations of Dimim [FeCl_4_]	Induced spin density	Stability and magnetic susceptibility	[65]
DFT calculations in N-vinyl-3-alkylimidazolium tetrahalogenidoferrate [VnC_1_im] [FeX] and N-vinyl-3-esterimidazolium [Vacim][FeCl_4_]	Interaction energies and the dipole moments	Surface tension and solubility	[66]
Interaction energies using SAPT0 DFT level in [C_2_C_1_im] [FeCl_4_], [C_4_C_1_im] [FeCl_4_], [C_4_C_1_im]-[FeBr_4_], and [C_4_C_1_im]_2_[SnCl_4_]	Electrostatic attractions	Surface charge and stability	[56]
DFT calculations using [C_4_C_1_im] [FeCl_4_] and bridge-chain [FeCl_5_] ^+^ and [FeCl_2_]^+^	DFT calculations together with X-ray absorption, Raman spectroscopy, and Dissociation reactions.	Atomic-scale structure and temperature effects	[66]
Imidazolium-based MILs and TEMPO-based radicals by DFT calculation of substituents like (N(CH_3_)_3_^+^ and OSO_3_^−^)	Bond order, electron Density difference and energy decomposition	Electrostatic interaction, micro-viscosity, and micro-polarity	[67]
Conductor-like Screening Model for Solvents (COSMO) and DFT methods in 1-alkyl-3-methylimidazolium bis (trifluoromethyl sulfonyl) imides, two 1-alkyl-1-methylpyrrolidinium bis (trifluoromethyl sulfonyl) imides, and two bis(1-alkyl-3-methylimidazolium) tetra-thio-cyanato-cobaltates	Isobaric and Isochoric heat capacity	Molecular volume, Density, Thermal conductivity, and Structural Composition	[68]
Force field models for ferrocenium-based MILs such as 1-alkyl-2,3,4,5,6,7,8,9- octamethylferrocenium bis (trifluoromethyl sulfonyl) imide ([CnFc] [NTf_2_],	Enthalpies of fusion and atom-atom interactions	Structural analysis, Charge densities, and Electrostatic interactions	[69]
Hybrid Reverse Monte Carlo (HRMC) calculations for [C_2_C_1_im]- [FeCl_4_] and [C_4_C_1_im][FeCl_4_]	Antiferromagnetic interactions	Crystallization	[70]
Non-polarizable force field and the universal force field (UFF) for [CnC_1_im][FeCl_4_]	Interactions between the external magnetic field and electrostatic interactions	Viscosity, self-diffusion Coefficients, density, electrical conductivity, and isobaric thermal expansion	[71]
MD simulation with radial distribution functions [C_4_C_1_im]- [FeCl_4_]	Hydrogen bonding interactions	Solubility, pH partition, and paramagnetism	[72]
DFT calculations for metal halide anions ([AlCl_4_]^−^ [CuCl_2_]^−^, and [FeCl_4_]^−^)	Hydrophobic interactions	Thermal stability and fluidity	[73]
DFT calculations for MILs [CnC_1_im]_2_[Co (SCN)_4_] (where *n* = 2, 4 and 6)	Absorption and desorption mechanisms	Structural analysis and electronic energies	[74]
DFT calculations for [Bmim^+^][FeCl₄^–^] catalyzed depolymerization of lignin	Hydrogen-bonding interactions	Electrochemical and catalytic properties	[75]

## Magnetic Ionic liquids assisted DNA extraction and drug delivery

Despite significant advances in nanomedicine, developing efficient, biocompatible, and targeted carriers for nucleic acids and hydrophobic drugs remains a challenge for the pharmaceutical industry. Magnetic drug delivery carriers have already shown promise as theragnostic tools due to their distinctive physicochemical properties, biodegradability, extended half-lives, and reduced toxicity compared to conventional gadolinium-based agents. In this context, MILs are emerging as a next-generation platform, created by incorporating transition metals such as Fe, Co, Ni, or Mn into ionic liquid structures. Their magneto-responsiveness, remarkable surface activity, and tunable wettability make them versatile for emulsification, catalysis, DNA extraction, and targeted drug delivery [76, 77].

Recent advances illustrate this potential. Deepak et al. synthesised transition-metal-based MILs (Ni, Co, Mn with [NTf₂⁻]), demonstrating high fluidity, strong lipophilicity, and water stability [78]. MILs demonstrate improved wettability, efficient binding, and enhanced stability at lower critical micelle concentrations (CMC) than traditional surface-active agents. Their unique self-assembly in magnetic fields makes them ideal for a range of applications, including drug delivery systems, chemical catalysis, nucleic acid extraction, nanomaterial synthesis, and environmental pollutant removal. Recently, a diverse range of cationic, anionic, non-ionic, and zwitterionic MILs has been developed, leading to new products and processes [79]. Gehlot and Singh et al. have created paramagnetic surface-active ionic liquids (PMSAILs) by introducing long alkyl-chain cations of imidazolium, pyridinium, or isoquinolinium, and bromotrichloroferrate(III) to impart paramagnetic properties. These surface-active ionic liquids have been examined for their aggregation characteristics using wettability measurements, conductivity measurements, and photon correlation spectroscopy. The interaction of the isoquinolinium cation of PMSAILs with genetic material in polar solvents showed adequate DNA compaction. The amassed understanding of DNA compaction under conditions analogous to those in actual cells may ultimately facilitate the design and construction of partially functional “artificial cells” with prospective uses in drug delivery systems, RNA-based therapy, and synthetic cell manufacture. The utilisation of PMSAIL_S_ as MRI contrast agents has also been investigated and demonstrated outstanding dual-mode T1 and T2 contrast in real-time practice, juxtaposed with the clinically accessible Gd-BOPTA (gadobenate dimeglumine)-based MRI contrast agent [80, 81]. However, key challenges remain. Toxicological profiles of MILs are underexplored, and their structural diversity complicates systematic evaluation. Biocompatibility, scalability, and regulatory acceptability must be addressed before translation into clinical practice. Notably, the diversity of MIL structures (cationic, anionic, zwitterionic) provides a rich toolbox but requires rational design frameworks for predictable performance. Xu, Lu et al. synthesised well-organised surfactant-DNA hybrid nanospheres comprising double-strand (ds) DNA and a cationic surface-active agent, coupled to the paramagnetic counterion [FeCl_3_Br]^−^. The unique properties of the FeCl3Br ion can condense DNA at elevated concentrations and facilitate the formation of structured nanospheres via accumulation, fusion, and coalescence. Cationic surface-active agents containing Br^–^ ion could not form nanospheres under the same conditions due to their loss of DNA compaction capability. A light-responsive, paramagnetic, cationic surface-active agent used to fabricate nanospheres enables the establishment of a dual-controlled drug-delivery system by applying an external magnetic field and alternating UV and visible light. The prepared nano-spherical delivery carrier exhibited enhanced drug absorption efficiency, sustained release, and biodegradability. These nanoplatforms offer opportunities for efficient magnetic-field-mediated targeted drug delivery with a photo-controlled drug-release profile. Such types of MILs may significantly contribute to the development of novel functional nucleic-acid-based nanoplatforms [82]. Wang, Ling, et al. developed an innovative and robust dual-responsive nano vehicle capable of efficiently integrating and utilising magnetic and glutathione (GSH)-reductive triggers to regulate payload delivery and enhance targeting precision. Paramagnetic fullerene (C60@CTAF) was synthesised using the [CTAB][FeCl_3_Br] (as counter ions of MILs) as a nano-vehicle by non-polar molecular aggregation. The enhanced dissolution and dispersity of fullerenes were achieved by optimal conjugation of C60 with [CTAB][FeCl3Br], which ultimately enhances DNA compaction. The DNA molecule within the nano-vehicles served as an electrostatic framework for loading the Doxorubicin molecule and as a crucial component in assembling the paramagnetic fullerene C60@CTAF/DNA. These complexes’ diminished agglomeration and modulated architecture result in uniform structures with enhanced integration of de-shielding and targeting functionalities, enhanced magnetic susceptibility, and external controllability via an external magnet. Further introduction of a hyaluronic acid coat addressed a simulated reductive extra-tumoral environment by effectively breaking disulfide bonds by GSH. It targeted drug delivery to liver cancer cell lines, making these cell lines promising candidates for novel nucleic acid-based drug carriers [83].

**Fig. 4:**
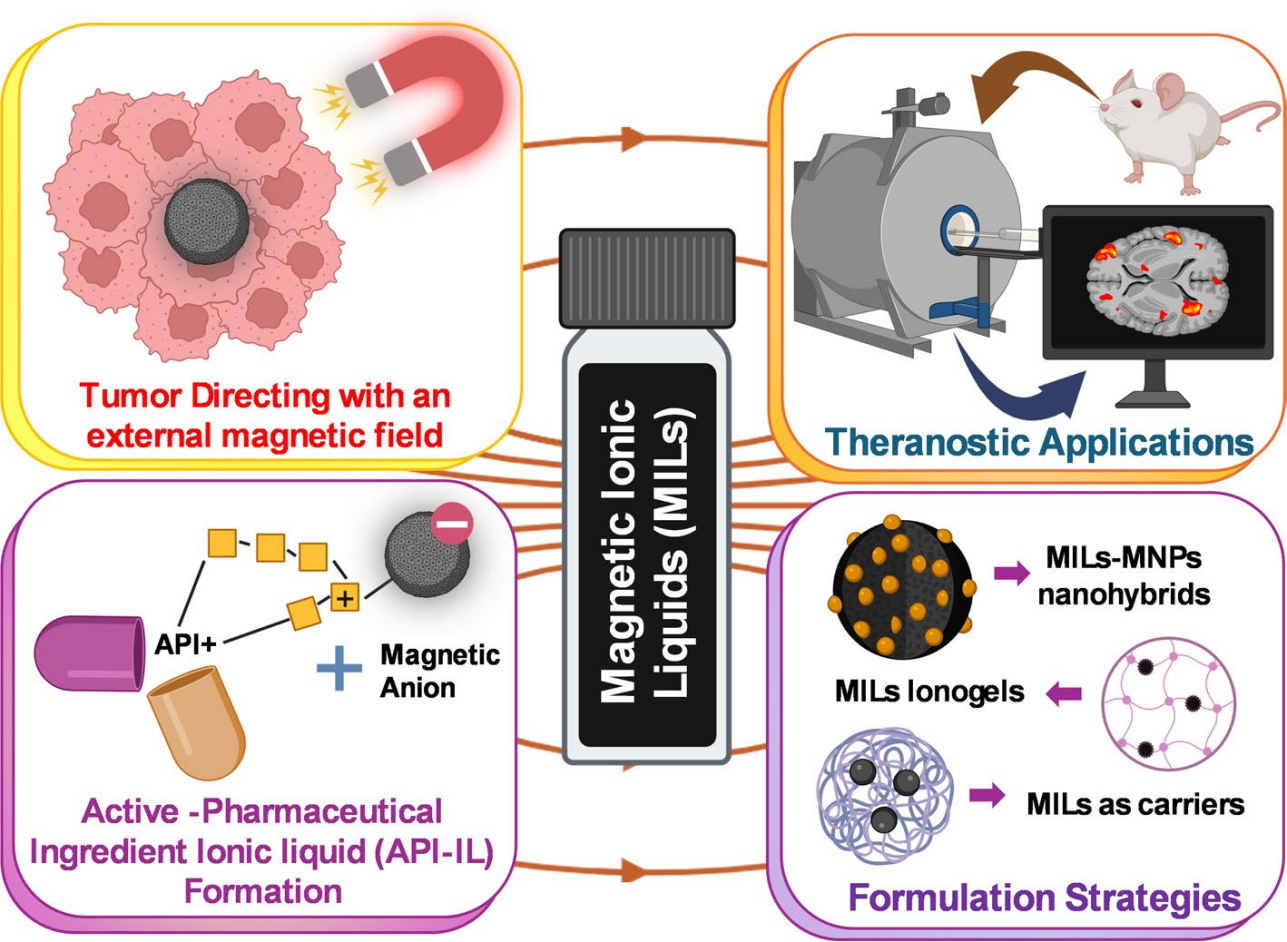
Various applications of MILs in drug delivery and biomedicine

Looking ahead, MILs represent more than incremental advances; they offer a paradigm shift toward multifunctional nanoplatforms that unite magnetic guidance, nucleic acid stabilisation, dual-mode imaging, and responsive drug release. Systematic efforts toward safety validation, scalable synthesis, and integration with RNA-based therapeutics and synthetic cell technologies could establish MILs as a clinically viable frontier in precision drug delivery. Table 2 compiles several applications of MILs in drug delivery and the extraction of genetic materials. Figure 4 illustrates various emerging applications of MILs in drug delivery and the biomedical field.

**Table 2:** Applications of promising Magnetic ionic liquids in drug delivery and the extraction of genetic materials

Type of ion in the MILs	Applications	Formulation	Ref.
Fullerene C60 conjugated [CTAB][FeCl_3_Br]	Enhanced solubility and targeting for anticancer agents	Magnetic Fullerene-DNA/Hyaluronic Acid Nano-vehicles	[83]
Trihexyl(tetradecyl)phosphonium tetrachloroferrate (III) ([P_6,6,6,14_^+^] [FeCl_4_^–^])	Nucleic acid extraction/DNA isolation	Surface-active Magnetic Ionic liquid	[84]
[N888Bn^+^] [FeCl_3_Br^−^]	Preservation of the biological activity of DNA	Hydrophobic magneto-responsive surfactant	[85]
((C1_2_H_25_)_2_N^+^(CH_3_)_2_[CeCl_3_Br]^−^)	Superior solvents for both hydrophilic and lipophilic drug molecules	DNA-lipid hybrid ferrofluid surfactants	[86]
bis[(trifluoromethyl)sulfonyl] imide [NTf_2_^−^] anion with Ni (II), Co (II), and Mn (II)	Reduced viscosity, elevated hydrophobicity, and hydrolytic resilience are used in chemical separations and drug solubilization.	Spherical or elongated micelles	[78, 87]
trimethylammonium trich loromonobromoferrate (C_16_H_33_(CH_3_)_3_N^+^ [FeCl_3_Br] ^−^	Dual controllable drug-delivery platform for anticancer agents	DNA-Surfactant Hybrid Nanospheres	[82]
(C_16_H_33_)_2_(CH_3_)_2_N^+^[FeCl_3_Br]	Magnetic surfactants for the Delivery/separation of biomolecules	Virus-shaped hybrid mixed assemblies of polyoxometalates	[88]
4-ethoxy4′-(trimethyl-aminoethoxy) azobenzene trichloromonobromoferrate (azoTAFe) with paramagnetic [FeCl_3_Br] − ion	Magnetic flux modulated target-specific delivery of DNA	Self-assembled micelles	[89]
Cetyltrimethylammonium with [FeCl_3_Br] −	Gene delivery and gene therapy	Rod-like micelles	[90]
[P_6,6,6,14_^+^] [FeCl_4_] and the [(C_8_)_3_BnN^+^] [FeCl_3_Br]	Plasmid DNA extraction	Polymerase chain reaction buffer with pDNA-enriched MIL	[91]
Cetyltrimethylammonium trichloromonobromoferrate (C_16_H_33_−N^+^ (CH_3_)_3_ Hydrophobic [FeCl_3_Br] ^−^	Molecular imprinting and modified drug or DNA release	Magnetic Bacillus-shaped bilayer Vesicles	[92]
Cetyl pentyl dimethylammonium trichloromonobromoferrate (C_16_C_5_DMA^+^[FeCl_3_Br]^−^)	Artificial Cell fabrication	Nano-transformer bilayer vesicle gels	[93]
[P_6,6,6,14_]^+^ [X(hfacac)_3_]^−^ where X = Co (II), Mn (II), or Ni (II).	Separation of non-enveloped viruses and viral genomic ssRNA	Magnetic ionic liquids Dispersions	[94]
Phenylpropyl guanidinium magnetic ionic liquid	Environmentally Friendly Selective Extraction of RNA	Nano-Drop	[95]
bis(1-butyl-3-methylimidazolium) tetrathiocyanatocobaltate ([Bmim]_2_[(SCN)_4_Co])	Tissue Engineering	Magnetically responsive magneto-ionic fibres	[96]

## Toxicity, biocompatibility, and regulatory aspects of MILs

The toxicity and biocompatibility of MILs remain inadequately characterized in in-vitro and in-vivo models. Initial toxicological studies of MILs, often containing imidazolium or cholinium cations paired with paramagnetic anions such as [FeCl₄^−^], [GdCl₆]³⁻, [CoCl₄]²⁻, or [MnCl₄]²⁻, reveal cytotoxic effects influenced by the metal type and cation structure. In human skin fibroblasts and Caco-2 cells, a lower IC₅₀ indicates greater toxicity after 24 h, with data showing [C₈MIM]₂[MnCl₄] (422.8 µM) as most toxic, followed by [C₈MIM]₂[CoCl₄] (541.8 µM), [C₈MIM]₃[GdCl₆] (678.9 µM), and [C₈MIM][FeCl₄] (1217 µM). Choline MILs display significantly reduced toxicity: [Choline-C1]₂[MnCl₄] at 1148 µM, [Choline-C1]₂[CoCl₄] > 1200 µM, with [Choline-C1][FeCl₄] and [Choline-C1]₃[GdCl₆] showing no toxicity within tested ranges (no IC₅₀ reached) [97]. This is mainly because first-row transition metals like Co and Mn tend to form labile complexes that are prone to hydrolysis, releasing free ions that generate reactive oxygen species (ROS) via Fenton-like reactions and disrupting cellular enzymes and mitochondria. The toxicity of [CoCl₄]⁻ and [MnCl₄]²⁻ MILs in fibroblasts and Caco-2 cells is linked to Co²⁺/Mn²⁺ ions interfering with calcium/magnesium homeostasis and electron transport. Mn particularly worsens neurotoxicity through dopaminergic pathway overload. Lanthanides such as Gd³⁺ exhibit high toxicity due to strong protein binding and nephrotoxic risks, despite stable coordination in [GdCl₆]³⁻. Some assays show toxicity surpassing that of Fe(III), which benefits from endogenous regulation via transferrin and ferritin, tighter ligand binding, and lower ROS production, resulting in an IC₅₀ exceeding 500 µM [98]. The cation or anion used to synthesize MILs significantly influences their toxicity profile. Imidazolium-based MILs tend to form micelles due to π-stacking and longer alkyl chains (C₈-C₁₀), facilitating membrane penetration and mitochondrial disruption, leading to cardiotoxic effects such as altered beat rate and amplitude at 10⁻⁵ M for C₁₀ variants. Toxicity increases by more than 700-fold from C₂ to C₁₀ due to increased lipophilicity, which promotes cellular uptake and ROS or mitochondrial damage; shorter chains (< C₄) tend to be less toxic. Cholinium-based MILs consistently exhibit lower toxicity than aromatic imidazolium types, aligning with general trends in which increased alkyl chain length and lipophilicity correlate with membrane and mitochondrial damage. The synergistic effect of hydrophobic ion pairs, combining lipophilic cations with labile, toxic anions like [CoCl₄]⁻, maximizes cytotoxicity through dual membrane disruption and ROS generation. Using bio-based cations such as cholinium and Fe anions mitigates these effects, resulting in safer profiles [99]. The key approach is designing pairs with low logP (< 2), high stability constants, and endogenous metals to facilitate clinical translation. In vivo toxicity data on MILs are scarce, as their applications have mostly been for trace-level analytical purposes where systemic exposure is minimal. Some emerging theranostic nanocomposites demonstrate tumour-specific effects in rodents without obvious short-term organ toxicity but lack detailed pharmacokinetic data. The broader literature on ILs warns of bioaccumulation risks, especially for hydrophobic, non-biodegradable ions, which are compounded in MILs by potential metal release and hydrolysis.

The regulatory environment for magnetic ionic liquids remains complex and largely non-specific, as MILs are currently regulated by general chemical regulations rather than specific guidelines. Depending on their use, MILs can be regulated as industrial chemicals, analytical reagents, pharmaceutical excipients, or active pharmaceutical ingredients, each requiring a different set of safety and quality information. The biggest problem stems from the highly customizable nature of MILs, in which slight modifications to cation-anion pairs can yield new compounds, requiring new registrations, toxicity testing, and environmental risk assessments for each new compound. Moreover, the paramagnetic nature of metal-based anions raises concerns about metal toxicity, persistence, and bioaccumulation, requiring thorough ecotoxicological and leaching tests. The lack of standardized naming conventions, the scarcity of long-term safety information, and the inability to predict structure-toxicity relationships further cloud the regulatory approval process. Furthermore, although MILs are often touted as “green” solvents because of their low volatility, regulatory agencies require extensive information about biodegradability, environmental impact, and proper disposal before approving such claims. Thus, the lack of globally harmonized guidelines, the high cost of data generation, and uncertainty regarding environmental fate conditions constrain the successful translation and commercialization of MIL-based technologies.

## Conclusions and prospects

Although research on MILs for drug delivery remains in its early stages, early reports demonstrate clear advantages over conventional organic solvents, particularly in improving the solubility of hydrophobic drugs and biomolecules. Their adjustable ferromagnetic properties enable compatibility with diverse polymers and biological systems, supporting applications in DNA extraction, permeation enhancement, and multifunctional delivery systems. The strategic selection of ferromagnetic ions enables MILs to simultaneously enhance solubility, stabilise biomolecules, and provide responsive functionality, positioning them as a versatile class of solvents for therapeutic development. MILs can be used as functional excipients that address challenges of drug targeting, stimuli-responsive nanomaterials, and solubilizers, and can serve as drug reservoirs.

Beyond drug delivery, MILs offer exciting opportunities in materials science and nanomedicine. For instance, [BMIm][FeCl₄] has been exploited for conductive polymerisation and the creation of functional nanomaterials, including poly(3-methyl thiophene) nanospheres (50–60 nm) [100], nanocomposites [101, 102], polypyrrole and poly(N-methyl pyrrole) nanotubes [103], nanofilms [104], and nano-sensors [105]. Parallel developments highlight their potential for diagnostic applications, in which methylimidazolium-type MILs exhibit resistive memory effects. [106, 107] The study of cation-anion interactions will help govern magnetic response performance, drug binding, stability, biocompatibility, and physiological toxic effects. Their ability to function as biosensors for early cancer detection and viral diagnostics underscores their biomedical promise. Furthermore, MILs have been proposed as MRI contrast agents, although limitations in terms of toxicity and colour contrast remain [13].

**Fig. 5:**
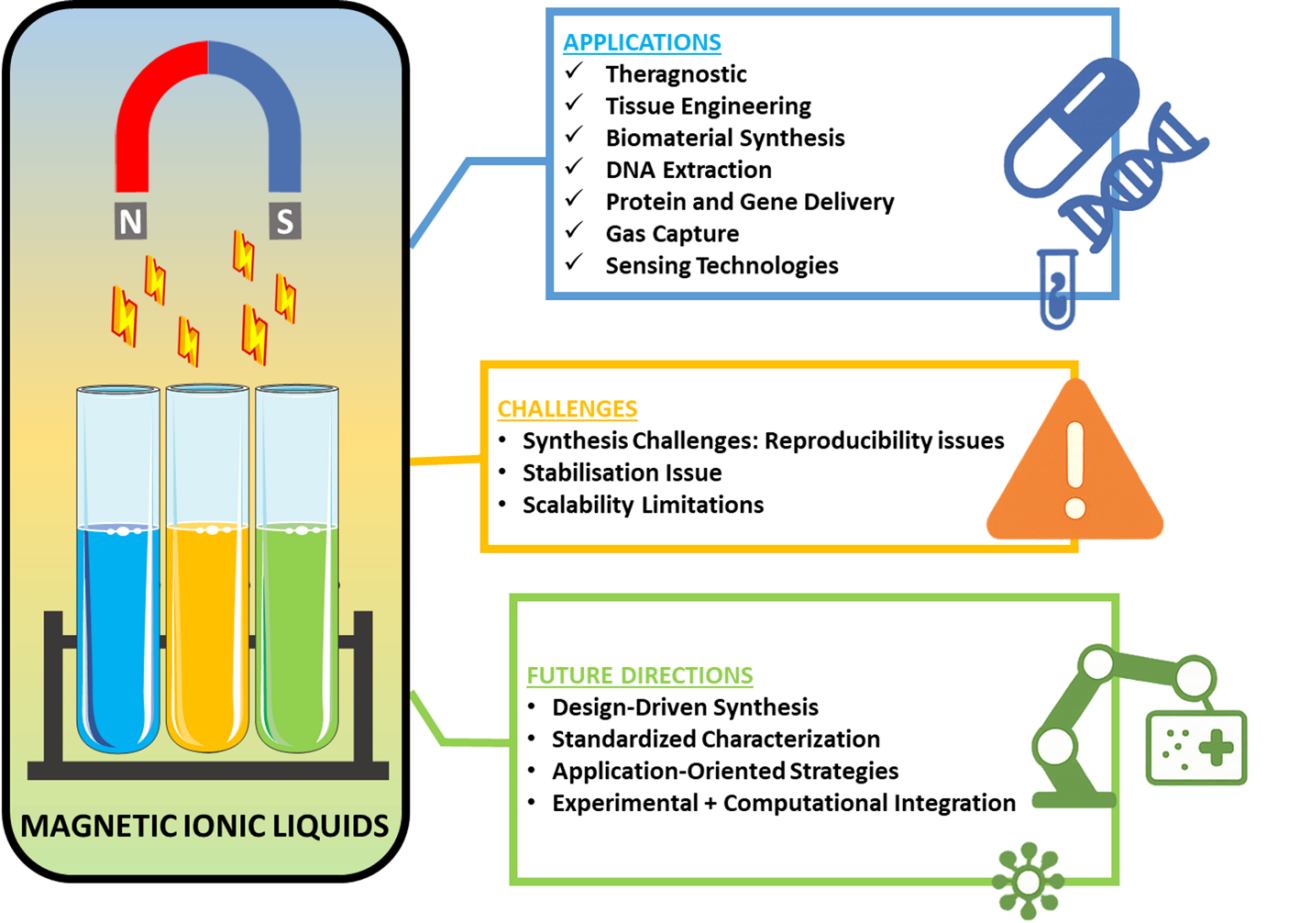
Use of new generation Computational modelling for designing MILs for desirable properties

Critical gaps persist, however. The toxicity, bioavailability, and biodegradability of MILs remain insufficiently characterised due to limited in vivo studies, raising concerns for clinical translation. Moreover, while their structural tunability is advantageous, it complicates systematic toxicity evaluation and regulatory acceptance. The magnetic behaviour of MILs will enable precise control over drug targeting when guided by an external magnetic field, thereby avoiding off-target effects and heat-related toxicity. MILs are multifunctional substances that, when used in conjunction with luminescent dyes, can serve as a theragnostic vector. Predictive computational modelling using tools such as DFT, CPMD, and conventional MD simulations will help standardize biodistribution, toxicity, and stability studies for MILs, which are essential to drive them from the laboratory to clinical practice. Looking forward, MILs could enable next-generation nanorobots, innovative nanocomposites, and biosensors capable of targeted delivery, disease monitoring, and controlled therapeutic release. Addressing safety, scalability, and integration with biomedical platforms through experimental and computational studies will be essential. Figure 5 summarizes the overall applications, challenges, and future directions of MILs.

In summary, MILs represent more than a niche innovation; they hold the potential to unify drug delivery, diagnostics, and nanotechnology into a single multifunctional platform. With focused research into their toxicology, biocompatibility, and engineering scalability, MILs may evolve from laboratory curiosities into clinically impactful tools for precision medicine and advanced healthcare.

## Data Availability

No datasets were generated or analyzed during the current study.

## Glossary

([Bmim]_2_[(SCN)_4_Co]): 1-butyl-3-methylimidazolium tetrathiocyanatocobaltate(II)
([P_6,6,6,14_^+^] [FeCl_4_^–^]): Trihexyl(tetradecyl)phosphonium tetrachloroferrate anion
[Bmim+][FeCl_4_^–^]: 1-butyl-3-methylimidazolium tetrachloroferrate
[C_8_MIM][FeCl_4_]: 1-octyl-3-methylimidazolium tetrachloroferrate(III)
[C_8_MIM]_2_[CoCl_4_]: Bis(1-octyl-3-methylimidazolium) tetrachlorocobaltate(II)
[C_8_MIM]_2_[MnCl_4_]: Bis(1-octyl-3-methylimidazolium) tetrachloromanganate(II)
[C_8_MIM]_3_[GdCl_6_]: Tris(1-octyl-3-methylimidazolium) hexachlorogadolinate(III)
[Choline-C1][FeCl_4_]: Cholinium tetrachloroferrate(III)
[Choline-C1]_2_[CoCl_4_]: Bis(cholinium) tetrachlorocobaltate(II)
[Choline-C1]_2_[MnCl_4_]: Bis(cholinium) tetrachloromanganate(II)
[Choline-C1]_3_[GdCl_6_]: Tris(cholinium) hexachlorogadolinate(III)
[CTAB]: Cetyltrimethylammonium bromide
[N888Bn^+^] [FeCl3Br^−^]: Benzyltrioctylammonium bromotrichloroferrate(III)
ACN: Acetonitrile
Ag: Silver
Au: Gold
CMC: Critical micelle concentration
Co: Cobalt
COSMO: Conductor-like Screening Model
CPMD: Car-Parrinello molecular dynamics
DCM: Dichloromethane
DFT: Density Functional theory
DMSO: Dimethyl sulfoxide
Dy: Dysprosium
Fe: Iron
Gd: Gadolinium
Gd-BOPTA: Gadobenate dimeglumine
ILs: Ionic liquids
MD: Molecular dynamics
MFe_2_O_4_: Metal ferrites
MIL: Magnetic ionic liquid
Mn: Manganese
Ni: Nickel
Pd: Palladium
Pt: Platinum
SAPT: Symmetry-adapted perturbation theory
